# Exploring the effects of high protein versus high fat snacks on satiety, gut hormones and insulin secretion in women with overweight and obesity: A randomized clinical trial

**DOI:** 10.1016/j.obpill.2025.100212

**Published:** 2025-09-27

**Authors:** Nahla Al-Bayyari, Maysoon Alhameedy, Razan Omoush, Hadeel Ghazzawi

**Affiliations:** a Department of Nutrition and Food Processing, Faculty of Al-Huson University College, Al-Balqa Applied University, Al-Salt, Jordan; b Department of Nutrition and Food Technology, Faculty of Agriculture, The University of Jordan, Amman, Jordan

**Keywords:** Greek yogurt, Gut hormones, Obesity, Overweight, Peanuts, Satiety

## Abstract

**Background:**

Nuts generally blunt the postprandial increases in glucose levels and increase satiety, while yogurt studies yield inconclusive results regarding post-meal hunger. This study investigated the effects of high-protein, and high-fat snacks, specifically Greek yogurt, and peanuts, on satiety, gut hormones, and insulin secretion in women with overweight and obesity. The hypothesis posited that peanuts would exhibit a more beneficial impact on satiety, gut hormones, and insulin levels compared to Greek yogurt.

**Methods:**

The two-arm parallel randomized trial involved fifty participants aged 30–40 years with a BMI between 25 and 35 kg/m^2^, randomly divided into peanut (n = 25) and Greek yogurt (n = 25) groups. After three days of adhering to 1200 Kcal diet, appetite sensations were gauged using a visual analog scale (VAS) upon arrival, and at 30- and 60-min post-snack. Pre- and post-snacking, plasma levels of cholecystokinin (CCK), Peptide Tyrosine-Tyrosine (PYY), Glucagon Like Peptide-1 (GLP-1), Ghrelin (GHRL), and insulin were analyzed.

**Results:**

Revealed that Greek yogurt induced a statistically significant increase in satiety 30 min after consumption and markedly elevated postprandial insulin levels compared to peanuts. Moreover, notable intergroup differences in postprandial insulin concentrations were observed in the Greek yogurt group. The peanut group had no significant alterations in PYY, GLP-1, CCK or GHRL levels. Pre-snacking, GHRL levels exhibited a positive association with abdominal circumference, weight, and fat mass, while CCK levels displayed a negative association with abdominal circumference, weight, and fat mass.

**Conclusion:**

Greek yogurt may enhance satiety and thus has the potential to positively influence body weight in individuals with overweight and/or obesity. Further research is required to elucidate appetite control mechanisms.

**Trial registration:**

The study was registered on ClinicalTrials.gov (No. NCT 04518930).

## Graphical abstract

Plain Greek yoghurt increased insulin levels and induced satiety but it has similar effect on GHRL, PYY, and GLP-1 compared to peanut snack. Peanut snack increased postprandial CCK compared to Greek yogurt. GHRL, Ghrelin; PYY, Peptide Tyrosine-Tyrosine; GLP-1, Glucagon Like Peptide-1; CCK, Cholecystokinin.

**Figure:**
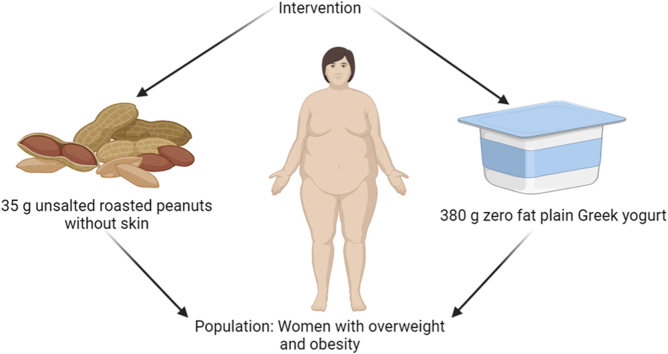

## 1 Introduction

Appetite regulation involves complex interactions between internal and external factors. Internal regulators, such as hormones like leptin, insulin, gut-derived peptides (GLP1, GIP, PYY), and ghrelin, play a crucial role in controlling hunger and satiety and external factors like environment and behavior, also contribute to energy intake regulation [1].

Peanuts, recognized for their protein, unsaturated fat, and fiber content, are considered a satiating snack, impacting post-meal satiety, and influencing hormones like CCK and PYY [[2], [3], [4], [5], [6], [7]]. Contrarily, yogurt studies show inconclusive effects on post-meal hunger compared to cheese or milk, with no significant variations in PYY or ghrelin concentrations [[8], [9], [10]]. Dairy product inclusion in children's diets demonstrates positive effects on food intake, satiety hormones, and appetite compared to sugar-sweetened beverages [11]. Notably, protein-to-calorie ratio exerts a stronger influence on *C*-Peptide, GLP-1, CCK, insulin, and insulin secretion rate compared to fat-to-calorie ratio [12].

Peanut consumption, especially after 12 weeks of low-carb dieting, impacts postprandial glycemia, showing increased levels of postprandial blood glucose with type 2 diabetes compared to almond consumers [13]. Nuts, in general, exhibit a blunt increases in the postprandial glucose levels and increased satiety [7]. Snacking studies reveal varied effects, with tree nuts and peanuts promoting heightened satiety, while peanuts, due to their energy density, exhibit high satiety value with minimal changes in overall energy balance [14,15]. A separate study highlights the satiety-enhancing effects of high-protein afternoon yogurt snacks, with Greek yogurt leading to decreased hunger, increased fullness, and delayed subsequent food intake compared to lower-protein snacks [16,17].

Cumulatively, the literature emphasizes the positive impact of both peanuts and yogurt on satiety and delayed hunger onset [3,[14], [15], [16], [17]]. Despite differing nutritional profiles, peanuts being rich in fiber and yogurt containing probiotics, both contribute to appetite regulation through the release of various gut hormones and insulin [16].

Notably, it becomes evident that the majority of studies have primarily focused on healthy adults or individuals with type 2 diabetes. These studies typically involve comparisons between higher protein versus low protein or high protein versus low fat snacks. However, the assessment of gut hormones alongside appetite and hunger perceptions in these studies has yielded contradictory results [7,13,16]. Additionally, there is a notable scarcity of studies comparing the effects of high fat versus high protein snacks on appetite control and satiety. Importantly, the snacks used in these comparative studies often do not include Greek yogurt and unsalted roasted peanuts, and the assessment of gut hormones is frequently omitted. Our study aims to address these gaps in the existing research by examining the impact of a plain high-protein Greek yogurt snack in comparison to a high-fat peanuts snack on hunger levels, satiety, appetite perception, insulin levels, and the secretion of gut hormones among women with overweight and obesity.

Given the provided information, our hypothesis assume that the consumption of high fat peanut snacks may lead to enhanced satiety, along with a more pronounced release of gut hormones and insulin, when contrasted with zero fat Greek yogurt. To test our hypothesis, a two-arm parallel randomized clinical trial was conducted on overweight and obese women.

## 2 Methods

### 2.1 Study design and participants

The study design utilized a parallel-arm intervention, employing a non-blind randomized clinical trial format with two distinct treatment groups. This study enrolled fifty-two healthy Jordanian women from a private nutrition clinic in Amman through randomization.

This study was conducted in accordance with the ethical guidelines outlined in the Declaration of Helsinki. The research procedures involving study participants received approval by the Institutional Research Board committee (No. 19/2021/180). Furthermore, the study was registered on ClinicalTrials.gov (No. NCT 04518930). Written informed consent was obtained from all individuals who participated in the study.

### 2.2 Inclusion and exclusion criteria

The study's inclusion criteria encompassed women aged between 30 and 40 years, with a body mass index (BMI) ranging from 25 to 35 kg/m^2^. The included age group comprises predominantly individuals with overweight and obesity attending the nutrition clinic, demonstrating high adherence to the study protocol. These individuals engaged in light exercise 1–3 times per week (activity factor 1.375) and adhered to a routine of consuming three regular meals, along with two daily snacks. They maintained a water intake of eight cups and ensured a sleep duration of 8 h. Additionally, participants were required to have no known allergies, particularly to nuts, especially peanuts and yogurt.

Conversely, women were ineligible for the study if they met any of the following conditions: age below 30 years or above 40 years, BMI below 25 kg/m^2^ or above 35 kg/m^2^, engaged in little or no exercise or engaged in moderate to heavy exercise [18], were pregnant or lactating mothers [19], had allergies to nuts, peanuts, yogurt, milk, or milk products, were using medications, hormonal therapy, supplements, oral contraceptives, or herbal/botanical products claimed to suppress appetite [[20], [21], [22], [23]]. Furthermore, exclusion criteria encompassed women with hormonal disturbances, including those in a menopausal state [24], and those within their menstrual cycle days or within one week prior, commonly experiencing hyperphagia due to elevated progesterone levels [25]. Additionally, individuals with chronic diseases and metabolic disorders such as cardiovascular diseases, diabetes, hypertension, kidney diseases, thyroid disorders, gastrointestinal diseases, polycystic ovary syndrome, or androgen disorders were excluded [[26], [27], [28], [29]]. Those following a weight-reducing diet, experiencing sleep disorders, or sleeping less than 8 h per day [30], and consuming less than 8 cups of water per day [31] were also excluded from the study.

### 2.3 Sample size determination

The required sample size for the study was determined using the formula [32]:

n = 2 ∗ (Zα + Zβ)^2^ ∗ SD^2^ ÷ δ^2^, where n represents the required number of participants in both the treatment and placebo groups. Zα and Zβ are the values from the standard normal distribution corresponding to specific confidence levels of 95 % and a two-sided α of 0.05 (Zα = 1.96) and a power of 80 % (Zβ = 0.84). SD refers to the standard deviation (pooled), while δ represents the estimated difference between the treatment and placebo groups.

Based on the above equation, the sample size calculation indicated that approximately 20 subjects were needed for each arm of the trial to detect a change of 1.76 μU/mL in insulin levels between the treatment and placebo groups, with 80 % power and 5 % significance. The standard deviation (SD) was assumed to be 2.1 [33]. Therefore, n = 2 ∗ (1.96 + 0.84)^2^ ∗ (2.1)^2^ ÷ (1.76)^2^ ≈ 22 participants per group.

To account for potential participant dropouts and to enhance the statistical power of the analysis, the number of participants was increased to 26 women per group.

### 2.4 Intervention

Two distinct interventional snacks were provided to the participants: plain Greek yogurt and peanuts. In the Plain Greek yogurt group (n = 25), each participant consumed 380g of plain Greek yogurt, equating to approximately 200 calories. In the peanut group, (n = 25) each participant consumed 35g of roasted, unsalted peanuts, providing approximately 200 calories.

The plain Greek yogurt (non-flavored/30g protein/13.5g carbohydrate/0g fat) was procured from the supermarket and stored in the refrigerator until serving. The peanuts (roasted, unsalted, with skin/8.4g protein/7.6g carbohydrate/17.5g fat) were purchased one day prior to the serving date to preserve their attributes. They were meticulously prepared in a safe and sterile manner, portioned into small transparent pouches, each containing 35g. These packed peanuts were then stored in a dry, room-temperature environment until the serving date. Table 1 shows the macro and micronutrients composition of the two interventional snacks. The intervention was provided to each participant upon arrival at the clinic based on pre randomization and they were asked to consume it at 1:00 p.m.

**Table 1:** Macro and micronutrients composition of one serving of plain Greek yogurt and unsalted skinned peanut snacks provided to women with overweight and obesity.

Variable	Greek yogurt	Unsalted peanut
Weight of the serving(g)	380	35
Calories (kcal)	200	200
Carbohydrate (g)	13.5	7.6
Dietary fiber	0.00	2.5
Fat (g)	0.00	17.5
Saturated fat (g)	0.00	2.5
Protein (g)	30.0	8.4
Vitamin D (mcg)	0.00	0.00
Sodium (mg)	136.4	0.00
Calcium (mg)	418	25
Iron (mg)	0.22	0.5
Potassium (mg)	535.8	225

An overview of the study design and procedures was illustrated in Fig. S1(Supplementary).

### 2.5 Randomization

The participants were assigned to two groups sequentially, following a predetermined randomization list generated by the principle investigator. “Peanut” and “Greek yogurt” were each.

written clearly in Arabic on 26 folded papers. The list was created by randomly selecting folded papers from a basket, each containing either the label "peanut," or "plain Greek yogurt". The groups were then identified as the peanut group, and the Greek yogurt Group based on the random selection of each participant.

All participants adhered to a 1200 kcal diet plan, including the same foods for breakfast, snack 1, lunch, snack 2, and dinner. This diet plan remained consistent for all participants over three consecutive days, except during the second visit, where specific snacks were provided based on the randomization list. To confirm adherence to the diet and ensure that the same foods were consumed at each meal, participants received a telephone call each morning. In addition, they were asked to send a message after each meal to confirm that the foods provided were fully consumed.

### 2.6 Data collection and assessment

#### 2.6.1 Pre-diet visit

During the initial visit, participants were provided with a three-day low-calorie diet menu totaling 1200 kcal. This menu included breakfast, snack 1, lunch, snack 2, and dinner, along with a follow-up sheet. They were instructed to adhere to the foods listed in the diet menu and to refrain from consuming peanuts or any peanut-based products, as well as Greek yogurt, during snack 1 and snack 2. The rationale for this approach is to mitigate the effect of prior food intake on participants' appetite and hormone levels before the intervention. Additionally, they were briefed on the necessity of daily follow-up via phone to monitor their compliance with the given instructions. Body composition analysis was performed using bioelectrical impedance analysis (Inbody 770). To categorize participants into groups, they were randomly allocated based on a pre-determined randomization list.

#### 2.6.2 Anthropometric and body composition analysis

Before the body composition analysis test, each participant visited the clinic after overnight fasting for a period of 10–12 h. The analysis was conducted using bioelectrical impedance technology, which automatically recorded several parameters, including weight, height, BMI, skeletal muscle mass, soft lean mass, fat mass, and abdominal circumferences. To ensure precise readings, participants were advised to wear lightweight clothing and remove any metal items, such as accessories, belts, money, and socks before the test.

#### 2.6.3 Post-diet visit

On the second visit, scheduled after three days of adhering to the 1200 Kcal diet, all participants arrived at the clinic at 1:00 p.m. Prior to their arrival, they had breakfast at home at 9:00 a.m. Upon arrival, a trained healthcare professional collected baseline blood samples from each participant.

At 1:00 p.m., participants in the plain Greek yogurt and peanut groups consumed their assigned snacks. Following snack consumption, a validated VAS questionnaire [34] was distributed to assess sensations and appetite levels at 0 min (1:00 p.m.), 30 min (1:30 p.m.), and 60 min (2:00 p.m.). Additionally, 1 h after their arrival, at 2:00 p.m., a second blood sample was collected from all participants. Throughout this period, participants were situated in a stress-free environment designed for relaxation and comfort, with soft music played to enhance their experience. No adverse events were reported by any of the participants following snack consumption.

#### 2.6.4 Appetite perceptions

Appetite sensation was evaluated using a validated VAS questionnaire [34], designed to measure subjective ratings of hunger, fullness, satiety, prospective food consumption, and desire to eat. The VAS questionnaire included questions assessing “how strong is your feeling of” hunger, fullness, and desire to eat or “how much food can you eat right now” with anchors of “not (much) at all” to “extremely/an extreme amount” [34].

Participants were instructed to indicate their sensations on the scale at various time points during the study: upon arrival, 30 min, and 60 min after consuming the snack. This allowed us to discern the impact of consuming Greek yogurt and peanuts on appetite perceptions over time. The decision to assess at 30 and 60 min was based on the rapid increase in gut hormones following food intake, with slight variations, their secretion typically peaks within 1–2 h postprandially [35]. The VAS questionnaire was self-administered, ensuring a convenient and straightforward assessment for the participants. ^34^

### 2.7 Biochemical analysis

The collected blood samples (baseline and after 60 min of intervention) were labeled with a unique code to ensure proper identification before being transported to the medical laboratory.

The blood samples were drawn into plain tubes containing separation gel and left undisturbed until coagulation occurred. After coagulation, serum was separated from the blood cells through centrifugation at 882×*g* for 20 min. The resulting serum was then carefully transferred into Eppendorf tubes and stored at −20 °C to preserve the integrity of the peptides for subsequent analysis. These storage procedures adhered to the recommendations outlined in studies conducted by Hallworth and Lobely [36,37].

All biochemical blood assays were conducted in duplicate, and the average of these duplicate measurements was utilized in the statistical analysis.

#### 2.7.1 CCK, PYY, GLP-1 and insulin analysis

The blood samples were sent to the laboratories of Aurum Biotechnology Company, where the researcher conducted the analysis following the instructions provided in the respective manuals. The analysis utilized the following ELISA kits: Human PYY ELISA kit (Lot No: 9680012101) (catalog no. RK02174; ABclonal, USA), Human GLP-1 ELISA kit (Lot No: 9680012107) (catalog no. RK09098; ABclonal, USA), Human CCK ELISA kit (Lot No: 1210528141) (catalog no. MBS770851; MyBioSourceA) and Human Insulin ELISA kit (lot No: 9680004240821) (Catalog. no. RK 00302; ABclonal, USA).

#### 2.7.2 Ghrelin analysis

The analysis of GHRL (ghrelin) was carried out using the Human GHRL ELISA kit (Lot No: 38401422) (catalogue no. MBS2602099; MYBioSource, USA). This kit utilizes the Competitive-ELISA method. The well plates used for the analysis were pre-coated with GHRL. In the reaction, the GHRL present in the sample or standard competed with a fixed amount of GHRL on the solid phase supporter for binding sites on the Biotinylated Detection Antibody specific to GHRL.

### 2.8 Primary and secondary outcomes

The primary outcomes are the changes in GHRL, PYY, GLP-1, CCK, and insulin levels, while the secondary outcomes include changes in satiety, fullness, hunger, desire to eat, prospective food consumption, and, ultimately, body weight.

### 2.9 Statistical analysis

The statistical analysis was conducted using SPSS version 25.0 (IBM SPSS Statistics for Windows, IBM Corporation). Before analysis, a nonparametric Kolmogorov-Smirnov test was employed to assess the normal distribution of all continuous variables. Descriptive statistics were used to assess the frequencies of categorical variables, while means ± standard deviations (SD) were calculated for continuous variables.

To determine differences between the means of normally distributed continuous variables in both the plain Greek yogurt and peanut groups, a Student t-test for independent samples was employed. Additionally, a paired sample *t*-test was used to assess variations in means of normally distributed variables before and after snack consumption in both groups. In instances where variables displayed significantly skewed distributions, medians were compared using the Mann-Whitney *U* test for independent samples, while paired samples were compared using the Wilcoxon Signed Rank test. A two-way repeated measures ANOVA was performed to test the main and interaction effects of Greek yogurt, peanuts, and time on appetite and insulin hormones. Additionally, the repeated measures procedure of the General Linear Model (GLM) was utilized to analyze the impact of plain Greek yogurt and peanuts on appetite perceptions over time.

Correlations between gut hormones, insulin, and anthropometric measurements were assessed using the Pearson two-tailed bivariate test for continuous variables. All p-values are nominal, two-tailed, and not adjusted for multiplicity. A significant level of p ≤ 0.05 was considered.

## 3 Results

### 3.1 General characteristics of the study subjects

**Fig. 1:**
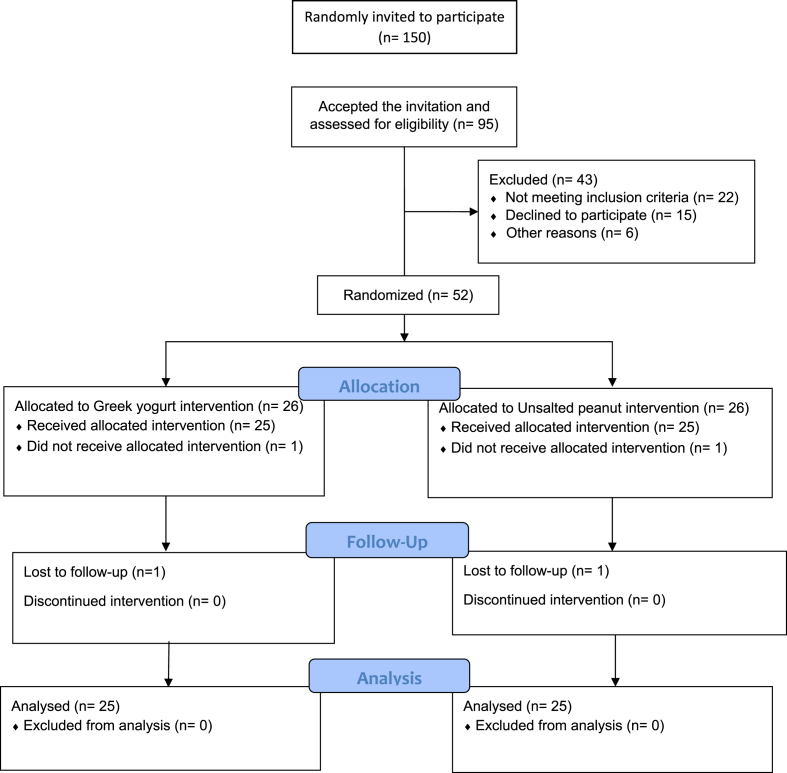
Selection of participants included in the study analysis.

Prior to the study, a total of one hundred and fifty women were randomly selected and invited to the clinic two weeks in advance. Out of the 150 randomly selected women, a total of 95 agreed to participate in the study after being informed about the study objective and protocol by the nutritionist. However, after further evaluation, 43 of them were excluded from the study due to various reasons. Finally, 52 participants met the inclusion criteria and were randomized into two different groups. Unfortunately, two participants were lost to follow-up as they did not attend the second visit (post-diet visit), resulting in a final analysis of 50 participants. The participant selection process for the trial is visually presented in Fig. 1. Of the participants, approximately three-fourths (72 %) were married and had less than three children (74 %). Moreover, no significant differences were observed between the two study groups concerning age, marital status, number of children, and BMI (Table 2).

**Table 2:** Comparing the age, BMI, and selected sociodemographic characteristics of women with overweight and obesity across Greek yogurt and unsalted peanuts groups (N = 50).

Variable	Range	Greek yogurt (n = 25) Mean ± SD	Unsalted peanuts (n = 25) Mean ± SD	P-value^
Age (Years)	10	35.2 ± 3.4	36.04 ± 3.5	0.397
BMI (kg/m^2^)	10	31.1 ± 3.2	30.8 ± 3.1	0.718

Variable	Total (N = 50) n (%)	Greek yogurt (n = 25) n (%)	Unsalted peanuts (n = 25) n (%)	P-value∗
Marital status				
- Married	36 (72.0)	20 (80.0)	16 (64.0)	0.173
- Single	14 (28.0)	5 (20.0)	9 (36.0)	
Number of children				
- < 3	37 (74.0)	18 (72.0)	19 (76.0)	0.500
- ≤ 3	13 (26.0)	7 (28.0)	6 (24.0)	

Data are presented as frequencies (n) and percentages (%).

∗ P-value ≤0.05 is significant for the Chi-square test.

^ P-value ≤0.05 is significant for the student t-test for independent samples.

P-values are not adjusted for multiplicity.

BMI: Body Mass Index.

### 3.2 Comparing anthropometric and biochemical measurements before intervention

Table 3 displays the comparison between the means ± SD of anthropometric and biochemical measurements for the Greek yogurt and peanut groups prior to the intervention. The results indicated that there were no significant differences (p > 0.05) between the two groups in terms

**Table 3:** Anthropometric and biochemical measurements for overweight and obese women before Greek yogurt or peanut intervention (N = 50).

Variable	Greek yogurt (n = 25) Mean ± SD	Peanut (n = 25) Mean ± SD	P-Value
Height (cm)	160.9 ± 5.7	158.9 ± 3.9	0.153
Weight (kg)	80.4 ± 7.6	77.7 ± 9.0	0.273
BMI (kg/m^2^)	^31.1^±3.2	^30.8^±3.1	0.718
Fat mass (kg)	34.8 ± 6.0	32.5 ± 7.2	0.235
Muscle mass (kg)	24.9 ± 2.5	24.8 ± 2.1	0.847
Soft lean mass (kg)	42.1 ± 4.5	41.7 ± 4.1	0.720
Fat free mass (kg)	46.1 ± 4.8	45.4 ± 4.3	0.614
Abdomen circumference (cm)	96.9 ± 7.1	95.4 ± 7.9	0.483
GHRL (ng/ml)	70.4 ± 7.4	70.6 ± 6.3	0.927
PYY (pg/ml)	124.7 ± 30.7	139.2 ± 37.4	0.139
GLP-1 (pg/ml)	172.6 ± 97.0	163.3 ± 94.9	0.732
CCK (pg/ml)	73.3 ± 16.7	84.6 ± 23.0	0.051
Insulin (pg/ml)	583.1 ± 182.4	457.0 ± 371.5	0.134

Data presented as means ±standard deviations (SD).

P-value ≤0.05 is significant for the student t-test for independent samples.

P-values are not adjusted for multiplicity.

BMI: Body Mass Index.

of anthropometric measurements, which included height, weight, BMI, fat mass, muscle mass, soft lean mass, fat-free mass, and abdomen circumference. Additionally, no significant differences (p > 0.05) were observed in their biochemical measurements, encompassing GHRL, PYY, GLP-1, CCK, and insulin.

### 3.3 Comparing appetite sensations scores over time

The results of the repeated measure analysis, conducted to examine the effect of time and snacks on the VAS appetite sensations in the study participants (N = 50), revealed a significant interaction effect between time and snacks group on fullness level [F(2,96) = 3.948, P = 0.023, η2 = 0.076] (Table 4).

**Table 4:** Results of repeated measures for the effect of time and snacks on the VAS appetite sensations for overweight and obese women (N = 50).

VAS (mm)	Snacks group			Time			Snacks group × Time		
	*F*	*P*	η^2^	*F*	*P*	η^2^	*F*	*P*	η^2^
Hunger	0.115	0.736	0.002	4.632	0.012	0.088	0.410	0.665	0.008
Fullness	0.380	0.540	0.008	4.897	0.009	0.093	3.948	0.023	0.076
Satiety	6.629	0.013	0.121	2.174	0.119	0.043	4.181	0.018	0.080
Desire to eat	0.609	0.439	0.013	13.336	<0.001	0.217	1.229	0.297	0.025
Prospective food consumption	0.051	0.822	0.001	14.279	<0.001	0.229	1.432	0.244	0.029

VAS: visual analog scale.

P ≤ 0.05 is considered significant for the GLM, repeated measures within and between subjects' effect.

η^2^: Partial Eta Squared.

**×:** Interaction.

P-values are not adjusted for multiplicity.

Furthermore, a significant main effect of snacks on satiety level was observed [F(1,48) = 6.629, P = 0.013, η2 = 0.121]. This implies that satiety varies depending on the type of snack intake by the participants. Pairwise comparison revealed higher satiety in the Greek yogurt group (26.5 ± 1.9), with a mean difference of −6.933, 95 % CI [−12.34, −1.52], P = 0.013. Additionally, a significant interaction effect between time and snacks group on satiety level was identified [F(2,96) = 4.181, P = 0.018, η2 = 0.080]. This indicates that the effect of time on satiety level differed between the Greek yogurt and peanuts groups (Table 4).

Moreover, all VAS appetite sensations (hunger, fullness, desire to eat, and prospective food consumption), except satiety, showed a significant main effect of time [F(2,96) = 4.632, P = 0.012, η2 = 0.088], [F(2,96) = 4.897, P = 0.009, η2 = 0.093], [F(2,96) = 13.336, P < 0.001, η2 = 0.217], [F(2,96) = 14.279, P < 0.001, η2 = 0.229], respectively (Table 4).

**Fig. 2:**
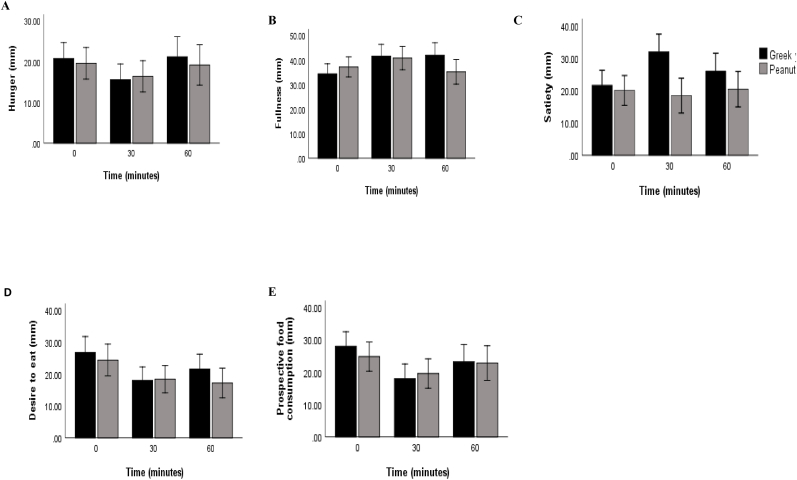
Appetite sensations were assessed using the VAS questionnaire, measuring (A) hunger, (B) fullness, (C) satiety, (D) desire to eat, and (E) prospective food consumption in millimeters at 0, 30, and 60 min following consumption of Greek yogurt and peanuts as snacks. The effect of the interaction between snack type and time on these sensations was evaluated using estimated marginal means within a general linear model. Results are presented as means ± S.E. Significant differences were observed in the estimated marginal means of (B) fullness and (C) satiety VAS sensations over time and snack groups. Conversely, no significant differences were detected in the estimated marginal means of (A) hunger, (D) desire to eat, and (E) prospective food consumption VAS sensations. VAS, Visual Analog Scale.

Fig. 2 illustrates notable variations in the estimated marginal means of fullness and satiety VAS sensations over time and snack groups. Of particular significance is the marked statistical difference in satiety observed at the 30-min between the Greek yogurt and peanut groups. In contrast, there were no significant differences detected in the estimated marginal means of hunger, desire to eat, and prospective food consumption VAS sensations.

### 3.4 Comparing gut hormones and insulin before and after intervention

Table 5 displays the mean plasma concentrations of GHRL, PYY, GLP-1, CCK gut hormones, and insulin for both study groups before and after the intervention. It also shows the main and the interaction effects of time and intervention on the plasma concentrations of these hormones and insulin.

**Table 5:** Plasma concentrations of ghrelin, cholecystokinin, peptide YY, glucagon-like peptide 1, and insulin in overweight and obese women before and after the intervention in both the plain Greek yogurt and peanut groups, and the two-way repeated measures ANOVA for the effect of time and intervention interaction (N = 50).

Variable	Greek yogurt group (n = 25)			Peanut group (n = 25)			2 × 2 ANOVA	
	Before Means ± SD	After Means ± SD	P-Value	Before Means ± SD	After Means ± SD	P-Value	Time	P∗
GHRL (ng/ml)	70.4 ± 7.4	69.0 ± 6.0	0.338	70.6 ± 6.3	69.9 ± 8.0	0.578	0.271	0.694
PYY (pg/ml)	124.7 ± 30.7	131.5 ± 26.9	0.198	139.2 ± 37.4	131.5 ± 24.8	0.226	0.916	0.078
GLP-1 (pg/ml)	172.6 ± 97.0	143.5 ± 42.1	0.126	163.3 ± 94.9	165.8 ± 77.0	0.914	0.374	0.291
CCK (pg/ml)	73.3 ± 16.7	73.2 ± 16.0	0.994	84.6 ± 23.0	88.6 ± 15.4	0.413	0.465	0.461
Insulin (pg/ml)	583.1 ± 182.4	1067.9 ± 529.3	<0.001	457.0 ± 371.5	503.6 ± 395.6	0.627	<0.001	0.002

Values are presented as means ± standard deviation (SD).

P < 0.05 is statistically significant for paired sample *t*-test.

P∗ ≤0.05 is statistically significant for the two way repeated measure ANOVA.

P-values are not adjusted for multiplicity.

GHRL: ghrelin, CCK: cholecystokinin, PYY: peptide YY, GLP-1: glucagon like peptide-1.

In the Greek yogurt group, a significant increase 60 min post-consumption (p < 0.001) was observed only in plasma insulin levels compared to prior Greek yogurt snack consumption. In the peanut group, no significant (p > 0.05) changes were noted in any of the plasma gut hormones or insulin after peanut snack consumption. Although CCK levels increased from 84.6 ± 23.0 to 88.6 ± 15.4 after peanut snacking, this change did not reach statistical significance.

The estimated marginal means of insulin concentrations revealed a statistically significant difference between the two study groups [F(1,48) = 17.146, P < 0.001]. In addition, there was a significant main effect of time, indicating that insulin levels increased from baseline to 60 min post-consumption across both groups [F(1,48) = 15.135, P < 0.001]. Importantly, a significant time × intervention interaction was observed [F(1,48) = 10.290, P = 0.002], demonstrating that the increase in insulin over time was greater in the Greek yogurt group compared with the peanut group.

### 3.5 Correlations between the gut hormones, insulin, and anthropometric measurements

Table S1 (supplementary) outlines the correlations between gut hormones, insulin, and anthropometric measurements. Prior to snack intake, baseline GHRL levels were positively correlated with several post-snack and anthropometric measures. Specifically, GHRL before snack intake was positively correlated with GHRL after snack intake (r = 0.528), insulin after snack intake (r = 0.338), abdominal circumference (r = 0.420), body weight (r = 0.430), and fat mass (r = 0.429). In contrast, baseline GHRL levels showed a significant negative correlation with CCK after snack intake (r = −0.333).

GHRL levels after snack intake were negatively associated with PYY levels prior to and after snack intake, soft lean mass, and height (r = −0.318, −0.447, −0.325, −0.279) respectively. PYY levels prior to snack intake displayed a negative correlation with CCK levels prior to snack intake (r = −0.337) and a positive correlation with PYY, GLP levels after snack intake, and soft lean mass (r = 0.565, 0.567, 0.287) respectively.

CCK levels prior to snack intake demonstrated negative associations with insulin, GLP-1 levels after snack intake, abdomen circumference, weight, muscle mass, and fat mass (r = −0.414, −0.319, −0.301, −0.470, −0.389, 0.344) respectively. Furthermore, CCK levels after snack intake exhibited a negative correlation with insulin levels after snack intake (r = −0.505).

## 4 Discussion

The study results indicated a notable impact of plain Greek yogurt snacks on satiety over time, as reflected in VAS ratings, compared to peanut snacks. However, there were no significant alterations observed in plasma levels of GHRL, PYY, GLP-1, or CCK before and after the consumption of either yogurt or peanut snacks. Significantly, only insulin levels showed a noteworthy increase following Greek yogurt consumption. Additionally, a substantial net difference in insulin levels between the groups was identified, with an increase in insulin observed in the Greek yogurt group. These findings contradict our initial hypothesis, suggesting that there is no evidence to supporting the idea that peanut snacks have a more favorable effect on satiety, gut hormone secretion, and insulin compared to Greek yogurt.

Assessing appetite involves evaluating various feelings, including hunger, fullness, satiety, desire to eat, and prospective food consumption. In the current study, when we assessed appetite in the minutes following the consumption of high protein (Greek yogurt) versus high fat (peanuts), we observed an increased feeling of satiety after 30 min. This finding supports the appetite-suppressing effect of protein shortly after consumption [16,17]. However, the larger volume of Greek yogurt consumed (380 g vs. 35 g for peanuts) and differences in micronutrient composition should not be overlooked. The potential contribution of gastric volume and stretch receptors (e.g., intraganglionic laminar endings) that activate vagal pathways to promote satiety [38] also highlights alternative mechanisms underlying the protein snack's impact on satiety. Additionally, the significant interaction of snacks by the time of intervention on satiety and fullness suggests that consuming snacks between meals has the potential to promote satiety and reduce overconsumption at subsequent meals [39].

Various mechanisms have been proposed to explain appetite suppression, including the involvement of gut hormones such as CCK, GLP-1, and PYY, which are suggested to induce satiety [39]. For instance, Onvani et al. [8] demonstrated that yogurt may lead to a reduction in post-meal hunger compared to cheese or milk, despite no significant changes in plasma PYY or ghrelin concentrations. Moreover, other researchers observed that the consumption of a Greek yogurt snack in the afternoon, containing 24 g of protein, significantly reduced hunger and promoted satiation when compared to high-fat snacks [17]. Our results are consistent with these previous research findings.

Changes in macronutrient composition and food components have been investigated for their potential role in appetite control, as they can impact hormones, metabolic pathways, gene expression, and appetite regulation [41]. In this study, the peanut snack had a high fat content, while the plain Greek yogurt snack contained zero fat, along with higher levels of carbohydrates and protein. Protein is recognized for its greater satiating effect compared to other nutrients, and a high-protein diet can stimulate the release of CCK, PYY, and GLP-1, as well as activate specific brain areas [42]. However, in our current study, the high-protein Greek yogurt snack Showed no significant changes in CCK, PYY, and GLP-1 levels, although it did significantly induce satiety at 30 min compared to the high-fat peanut snack. This contradictory result may be attributed to our omission of assessing hormone level changes at 30 min post-consumption, or at 30-min intervals over a longer duration. Other studies have reported increased satiety after consuming 15 g of whey protein alongside a mixed macronutrient breakfast, as well as elevated PYY levels and reduced hunger following a high-protein, moderate-carbohydrate diet compared to a moderate-protein, high-carbohydrate diet [[43], [44], [45]]. The variation in results may be attributed to the longer duration required for peanuts to induce satiety and to differences in macronutrient composition. Moreover, ongoing research on the satiating capacity of plant-derived and animal-derived proteins, with the inclusion of plant-derived proteins, is showing promising results [46]. Additionally, exploration of differences in protein satiation between men and women is underway, with some studies indicating a potentially stronger satiating effect in men [47].

Previous studies examining GLP-1 secretion in response to meals containing carbohydrates, fats, or proteins have produced conflicting results [40,48]. While some studies have observed a decrease in GLP-1 secretion in response to fats compared to carbohydrates, the findings have been inconsistent concerning protein-induced GLP-1 secretion [48]. However, emerging evidence suggests that protein, particularly from dairy sources, can stimulate GLP-1 release. For instance, a narrative review highlighted that foods containing both protein and calcium, such as dairy products, have been shown to enhance GLP-1 release. Specific amino acids like glutamine, phenylalanine, arginine, and tryptophan have also been identified as potent stimulators of GLP-1 secretion [49]. Furthermore, a study comparing high-protein meals to other macronutrient compositions found increased secretion of GLP-1 and PYY, indicating a potential appetite-suppressing effect [50]. These findings underscore the importance of considering protein intake in dietary strategies aimed at modulating GLP-1 levels and appetite regulation. After considering the role of GLP-1 in promoting satiety, it is also important to look at ghrelin, which works in the opposite direction by stimulating hunger. While our study did not observe a significant reduction in ghrelin following snack consumption, other research has reported decreases in ghrelin after intake of snack bars enriched with whey protein and polydextrose [51,52]. However, these findings should be interpreted with caution, as ghrelin levels are highly dynamic and fluctuate naturally throughout the day depending on hunger and satiety status, making direct comparisons between fasting and postprandial levels difficult [53]. Taken together, this highlights the complexity of ghrelin's role in appetite regulation and underscores the need for careful timing and repeated measurements when studying its response to different foods.

Regarding insulin levels, most studies examining the effects of protein intake on serum insulin have reported no significant changes following whey protein consumption or high-fiber diets [51,[54], [55], [56]]. The absence of differences in hormones related to hunger and satiety, including CCK, GLP-1, PYY, GHRL, insulin, and leptin, between nut consumption and the control meal suggests that the mechanism by which nuts enhance satiety may not occur over a short period of time [51].

In our study, we observed an increase in postprandial plasma insulin levels in the yogurt group compared to the peanut group although this is not usually a favorable outcome, especially since we do not have baseline data on beta-cell function. Acute trials have demonstrated that milk proteins can mitigate the postprandial increase in blood glucose when consumed with high glycemic response foods [57]. Ostman et al. [57] reported that the addition of fermented milk (yogurt) to a breakfast with a high glycemic index significantly blunt the postprandial increases in glucose levels and insulinemia. Dairy snacks, like yogurt, containing bioactive proteins such as casein and whey protein [58], have unique macronutrient compositions that can influence insulin levels through various mechanisms, including increased release of GLP-1 and altered hepatic insulin extraction [[58], [59], [60]]. However, the specific components of the dairy food matrix responsible for the substantial increase in circulating insulin are not fully understood, although the rise in insulin has been correlated with the appearance of certain amino acids in the bloodstream [[61], [62], [63]]. Unfortunately, the amino acid profiles of the participants after snacking were not assessed in the current study.

Previous studies have explored the effects of various types of nuts on appetite and have suggested that nuts can enhance postprandial satiety [3,4,64]. Nuts are rich in protein, fiber, and phenolic compounds. When consumed with other foods, they exhibit a low glycemic index, potentially contributing to prolonged satiety until the next meal [3,65]. Consumption of nuts has been shown to suppress hunger and increase the sense of fullness in individuals with normal weight; however, in individuals with overweight or obesity, these effects were observed but not statistically significant [3]. Nevertheless, one study reported no significant effect of peanut consumption on satiety [66], which is consistent with our findings. In contrast, another study found that peanuts significantly influenced both satiety and energy intake [67]. These contradictory findings may be explained by differences in study design, portion size, duration of intervention, or characteristics of the study population, all of which can influence satiety responses.

Weight status may influence the impact of nuts on satiety, as individuals with overweight or obesity tend to experience less suppressed hunger, diminished sense of fullness, and increased energy intake after consuming nuts compared to those normal weight individuals [3]. The perception of hunger and satiety in individuals with obesity can be influenced by various factors, including hormonal changes [68]. However, further research is needed in this area to reach more conclusive findings.

## 5 Limitations

The primary strength of this study is its clinical trial design and the use of the VAS to assess appetite sensations, alongside the measurement of various gut hormones and insulin. Although the main limitations are the small sample size, the parallel group design, the study lacks a placebo or control group and does not include biochemical analysis of blood glucose or other hormones, such as amylin, leptin, and adiponectin, which are known to influence satiety and hunger. The study also did not measure insulin and gut hormone levels at 30 min post-intervention, only at baseline (0 min) and 60 min post-intervention and the results cannot be generalized to the entire population. This is because the study population was limited in size and demographic diversity, including age, gender, and BMI, and therefore may not represent broader populations. Although peanuts are botanically legumes rather than tree nuts, in everyday eating patterns and much of the nutrition literature they are often considered alongside tree nuts. This is due to their similar nutrient profile, culinary use, and potential health effects [69]. Still, this distinction is worth keeping in mind when interpreting our results.

## 6 Conclusions

This study contributes to our understanding of the effects of yogurt and peanut snacks on appetite-related factors and satiety. Specifically, the Greek yogurt snack demonstrated a significant satiating effect and led to increased postprandial insulin secretion compared to the peanut snack. This suggests that Greek yogurt could offer advantages in weight management by delaying the onset of subsequent food consumption. Additionally, its impact on postprandial glycemia, whether beneficial or harmful, remains uncertain as we were unable to assess it due to a lack of glucose indices data. Nevertheless, further research is warranted to delve into the underlying mechanisms of appetite control and to investigate how varying macronutrient compositions and food components influence appetite regulation, insulin, and blood glucose indices. Furthermore, future studies should include broader populations to enhance external validity and improve generalizability.

### 6.1 Key takeaway clinical messages

• Snacking on Greek yogurt increased satiety at 30 min compared to peanut snacking.
• Plasma insulin levels increased after Greek yogurt snacking compared to peanut snacking.
• Ghrelin increases with weight, fat, and abdomen size per correlation analysis.
• Cholecystokinin drops with weight, fat, and abdomen size per correlation analysis.
• Snacking on Greek yogurt may promote satiety and aids in energy management.

## Ethics review

This study was conducted in accordance with the ethical guidelines outlined in the Declaration of Helsinki. The research procedures involving study participants received approval by the Institutional Research Board committee (No. 19/2021/180). Written informed consent was obtained from all individuals who participated in the study.

## Author contributions

**Nahla Al-Bayyari:** Conceptualization, methodology, supervision, resources, formal analysis, writing-review & editing. **Maysoon Alhameedy:** Conceptualization, investigation, methodology, resources, funding acquisition, writing-original draft. **Razan Omoush:** Writing-review & editing, data curation. **Hadeel Ghazzawi:** Supervision, Conceptualization, methodology, Writing-review & editing. All authors critically revised the manuscript, and approved the final manuscript.

## Trial registration

The study was registered on ClinicalTrials.gov (No. NCT 04518930).

## Declaration of artificial intelligence (AI) and AI-assisted technologies

The authors did not use AI tools during the preparation of this work.

## Funding

This work was funded by the 10.13039/501100021772Deanship of Scientific Research at the University of Jordan (grant number 19/2021/187). The University of Jordan had no role in the design, analysis or writing of this article.

## Declaration of competing interest

The authors declare the following financial interests/personal relationships which may be considered as potential competing interests:Maysoon Alhameedy reports financial support was provided by The University of Jordan. If there are other authors, they declare that they have no known competing financial interests or personal relationships that could have appeared to influence the work reported in this paper.

## Abbreviations

Body Fat Percentage (BFP); Body Mass Index (BMI); Cholecystokinin (CCK); Ghrelin (GHRL); Glucose-Dependent Insulinotropic Polypeptide (GIP); Glucagon Like Peptide-1 (GLP-1); Peptide; Tyrosine-Tyrosine (PYY); Visual Analog Scale (VAS).
