# Supplementary material for: Exploring the effects of high protein versus high fat snacks on satiety, gut hormones and insulin secretion in women with overweight and obesity: A randomized clinical trial
Source: Obes Pillars. 2025 Sep 27;16:100212. doi: 10.1016/j.obpill.2025.100212 (PMC12513319; doi:10.1016/j.obpill.2025.100212)
Supplement: Multimedia component 1 [file mmc1.docx]

**Post-diet visit (Second visit)**

- All participants arrived at 1:00 pm, and basal blood samples were withdrawn upon arrival.
- All participants completed the VAS questionnaire at 0 time of intervention.
- All participants consumed their snack according to their randomization.
- All participants completed the VAS questionnaire again after 30 and 60 minutes of snack consumption.
- Blood samples were collected again after 60 minutes of snack consumption.

**Pre-diet visit (Initial visit)**

- All participants arrived fasting for 10-12 hours
- Anthropometric measurements and body composition analysis were performed
- All received a three-day low calorie diet totaling 1200 Kcal

**peanut group**

Received one serving (35 g, 200 Kcal) unsalted roasted peanuts

**Greek yogurt group**

Received one serving (380g, 200Kcal ) zero fat Greek yogurt

**Study participants**

**Randomization into two study groups**

**Figure S1:** Overview of the study design and procedures
