# Supplementary material for: Exploring the effects of high protein versus high fat snacks on satiety, gut hormones and insulin secretion in women with overweight and obesity: A randomized clinical trial
Source: Obes Pillars. 2025 Sep 27;16:100212. doi: 10.1016/j.obpill.2025.100212 (PMC12513319; doi:10.1016/j.obpill.2025.100212)
Supplement: Multimedia component 2 [file mmc2.docx]

**Table S1** Correlations between the gut hormones, insulin, and anthropometric measurements among the overweight and obese women in the study (N = 50).

| Variable | GHRL before | GHRL  after | PYY before | PYY after | CCK before | CCK after | Insulin before | Insulin after | GLP before | GLP after | Soft lean mass | Fat  Free  mass | AC | Weight | Height | Muscle mass |
| --- | --- | --- | --- | --- | --- | --- | --- | --- | --- | --- | --- | --- | --- | --- | --- | --- |
| GHRL before | 1 |  |  |  |  |  |  |  |  |  |  |  |  |  |  |  |
| GHRL after | .528** | 1 |  |  |  |  |  |  |  |  |  |  |  |  |  |  |
| PYY before | -.238- | -.318-* | 1 |  |  |  |  |  |  |  |  |  |  |  |  |  |
| PYY after | -.256- | -.447-** | .565** | 1 |  |  |  |  |  |  |  |  |  |  |  |  |
| CCK before | -.267- | -.104- | -.373-** | -.256- | 1 |  |  |  |  |  |  |  |  |  |  |  |
| CCK after | -.333-* | -.266- | .189 | .109 | .534** | 1 |  |  |  |  |  |  |  |  |  |  |
| Insulin before | -.097- | .040 | .030 | .121 | -.091- | -.059- | 1 |  |  |  |  |  |  |  |  |  |
| Insulin after | .338* | .269 | -.141- | -.046- | -.414-** | -.505-** | .293** | 1 |  |  |  |  |  |  |  |  |
| GLP before | -.176- | -.142- | .143 | .040 | -.190- | -.173- | -.104- | .091 | 1 |  |  |  |  |  |  |  |
| GLP after | .217 | -.002- | .567** | .248 | -.391-** | -.026- | -.122- | -.091- | .165 | 1 |  |  |  |  |  |  |
| Soft lean mass | -.072- | -.325-* | .287* | .393** | -.234- | -.030- | -.035- | .053 | .019 | .189 | 1 |  |  |  |  |  |
| Fat free mass | -.028- | -.296-* | .273 | .383** | -.267- | -.054- | -.022- | .077 | .017 | .185 | .997** | 1 |  |  |  |  |
| AC | .420** | -.073- | .046 | .156 | -.301-* | -.071- | -.186- | .176 | .105 | .162 | .213 | .272 | 1 |  |  |  |
| Weight | .430** | .044 | .105 | .179 | -.470-** | -.115- | -.161- | .217 | -.094- | .116 | .548** | .604** | .803* | 1 |  |  |
| Height | -.254- | -.279-* | .140 | .076 | -.238- | -.022 | .006 | .018 | .046 | -.097- | .482** | .491** | .051 | .339* | 1 |  |
| Muscle mass | .152 | -.092- | .193 | .167 | -.389-** | -.193- | .034 | .109 | -.114- | .090 | .761** | .768** | .049 | .552** | .598** | 1 |
| Fat mass | .429** | .108 | .023 | .120 | -.344-** | -.026- | -.210- | .198 | -.048- | .099 | .221 | .285* | .933** | .881** | .062 | .093 |

Correlation is significant at * p < 0.05 , ** p < 0.01, *** p < 0.001

P-values are not adjusted for multiplicity

GHRL: ghrelin, CCK: cholecystokinin, PYY: peptide YY, GLP: glucagon like peptide 1, AC: Abdomen circumference
